# Antibody-free quantification of seven tau peptides in human CSF using targeted mass spectrometry

**DOI:** 10.3389/fnins.2015.00302

**Published:** 2015-09-01

**Authors:** Pauline Bros, Jérôme Vialaret, Nicolas Barthelemy, Vincent Delatour, Audrey Gabelle, Sylvain Lehmann, Christophe Hirtz

**Affiliations:** ^1^Laboratoire de Biochimie et de Protéomique Clinique - Institut de Médecine Régénérative et Biothérapies, Centre Hospitalier Universitaire de MontpellierMontpellier, France; ^2^Laboratoire National de Métrologie et d'Essais (LNE)Paris, France; ^3^Centre Mémoire Ressources Recherche, Centre Hospitalier Universitaire de Montpellier, Hôpital Gui de Chauliac, Université Montpellier IMontpellier, France

**Keywords:** Alzheimer's disease, tau protein, human cerebrospinal fluid, LC-MS/MS, quantitative proteomic, triple quadrupole

## Abstract

Tau protein concentration in cerebrospinal fluid (CSF) is currently used as a sensitive and specific biomarker for Alzheimer's disease. Its detection currently relies on ELISA but the perspective of using mass spectrometry (MS) to detect its different proteoforms represents an interesting alternative. This is however an analytical challenge because of its low concentration in the CSF, a biological fluid collected in small volume by lumbar puncture, and with a high structural heterogeneity. To overcome these issues, instead of using immunocapture as previously done, we rather relied on an original two steps pre-fractionation technique of CSF: perchloric acid (PCA) followed by micro solid phase extraction (μSPE). We could then measure seven tau trypsic peptides by Multiple Reaction Monitoring (MRM) on a triple quadrupole mass spectrometer. Quantification was performed using isotopically labeled ^15^N- recombinant tau protein as internal standard and validated using CSF pools with low, medium, or high tau concentrations (HTCs). Repeatability, intermediate precision, linearity, limit of quantification (LOQ), and recovery were calculated for the different peptides. This new MRM assay, which allowed for the first time CSF tau protein quantification without immunocapture, has important potential application to follow tau metabolism in both diagnostic and therapeutic research.

## Introduction

Alzheimer's disease (AD) is the most common form of dementia, a general term encompassing loss of memory and cognitive functions interfering therefore with activities of daily living. With 35 million of patients worldwide, AD represents 50–80% of cases of dementia and is reaching epidemic proportions in industrialized countries mainly because of the aging of the population. At neuro-pathological level, AD is characterized by the presence in the brain parenchyma of amyloid plaques and hyperphosphorylated tau (p-tau) proteins aggregated into neurofibrillary tangles (Gabelle et al., 2010). Tau protein is synthesized by a single microtubule-associated protein tau (MAPT) gene (chromosome 17q21) in humans (James et al., 2015) mainly expressed in neurons (Perrin et al., 2009). Tau protein is known to bind neuronal microtubules, promote their assembly, and stabilize them (Drechsel et al., 1992). The hyperphosphorylation occurring in case of AD caused its release from microtubules, destabilizing the axons, and triggering neuronal death (Sergeant et al., 2005). Quantification of tau in cerebrospinal fluid (CSF) is currently used as a sensitive and specific biomarker for AD diagnosis (Andreasen and Blennow, 2005). However, the full quantification of tau in CSF remained an analytical challenge. In fact, this protein has 6 different isoforms (ranging from 352 to 441 amino acids), and is subject to many different post-translational modifications like phosphorylation, glycosylation, and oxidation (Hernandez and Avila, 2007). Moreover, tau is present at very low concentration (Sjögren et al., 2001; Blennow and Vanmechelen, 2003) in CSF which is a highly complex matrix.

Currently, CSF tau quantification is performed in clinical daily routine by immunoassays (ELISA). If immunoassays are sensitive enough to detect low CSF tau concentrations, they present some boundaries: poor linearity, lack of specificity in regards to multiple tau proteoforms and no multiplexing. This is the reason why mass spectrometry (MS) is an interesting alternative in this context. In 2014, McAvoy et al. (2014) has published a quantitative LC-MS/MS method to measure tau in CSF with a sample preparation based on a immunoaffinity. Barthelemy et al. recently quantified 22 tau peptides by high resolution MS (Barthelemy, under review). Since this equipment is very rarely available in clinical laboratories, we used the same sample preparation protocol but quantification was performed on a triple quadrupole mass spectrometer. In this work, we describe the set-up of the first multiplex targeted MS quantification of tau in CSF without the need of immunocapture. The workflow includes a CSF protein precipitation and a solid phase extraction followed by a trypsic digestion. Seven tau proteotypic peptides located in different positions of the protein could be quantified by Selected Reaction Monitoring. This new method has important potential applications to follow tau metabolism in both diagnostic and therapeutic research.

## Materials and methods

### Reagents

Perchloric acid (PCA), trifluoroacetic acid (TCA), and hydrogen peroxide (H_2_O_2_) were purchased from Sigma Aldrich (Saint Quentin Fallavier, France). Ammonium bicarbonate and trypsin were obtained from Fluka-Sigma Aldrich (Saint Quentin Fallavier, France) and Promega (Charbonnieres, France), respectively. Water, formic acid (FA), methanol (MeOH) were all ULC-MS grade and purchased from Biosolve (Dieuze, France). Normal Goat serum was obtained from Clinisciences (Nanterre, France). Protein LoBind tube 1.5 mL and Deepwell plate 96/500 μl Protein LoBind were purchased Eppendorf (Le Pecq, France). Oasis HLB μElution Plate 30 μm, was obtained from Waters (Guyancourt, France). Polypropylene vials and Zorbax 300 SB-C18 1 × 150 mm 3.5 μm were both purchased from Agilent Technologies (Santa Clara, CA, USA).

#### Human CSF samples and ELISA CSF Tau quantification

CSF samples were obtained from patients followed-up by the Montpellier neurological and Clinical Research Memory Centers (CMRR) for cognitive or behavioral disorders. They gave their informed consent for research and for the storage of their sample in an officially registered biological collection (#DC-2008-417) of the certified NFS 96-900 biobank of the CHRU of Montpellier (Ref: BB-0033-00031, www.biobanques.eu). CSF was collected in polypropylene tubes under standardized conditions, preferably between 9:00 a.m. and 1:00 p.m., to minimize the influence of diurnal variation. Each CSF sample was sent to the local laboratory within 4 h after collection and was centrifuged at 1000 g for 10 min at 4°C. CSF was aliquoted in polypropylene tubes of 1.5 mL and stored at −80°C until further analysis. Tau quantification was performed by ELISA InnoTest Tau from Fujirebio diagnostics following manufacturer's instructions.

#### Standards and samples preparation

##### Preparation of ^14^N and ^15^N recombinant tau protein (441) standards

^14^N and ^15^N recombinant tau protein (441) were obtained from Dr. Guy Lippens (UMR 8525, Lille Pasteur Institute, France). Lyophilized standards were resuspended at 1 mg/mL with ammonium bicarbonate 50 mmol/L. The solution was then aliquoted into 50 μL in LoBind tubes and stored at −80°C until use. Concentration of ^14^N and ^15^N tau primary calibrators was determined by amino acid analysis. Ten calibration standards were prepared gravimetrically using an analytical balance model Sartorius CPA224S-OCE (Sartorius Goettingen, Germany) by adding a fixed amount of ^15^N tau to variable amounts of ^14^N tau. Standards were diluted with 50 mmol/L ammonium bicarbonate and 1 mmol/L BSA and then further diluted in 0.5% goat serum so as to reach final concentration of 5 ng/mL for ^15^N tau while that of ^14^N tau ranged from 0.3 to 32.1 ng/mL.

Series of 8–10 human CSF samples with tau concentrations determined by ELISA were mixed to obtain 3 CSF pools with Low Tau Concentration (LTC), Medium Tau Concentration (MTC), and High Tau Concentration (HTC).

##### Precipitation, μSPE extraction and protein digestion

**Figure 1:**
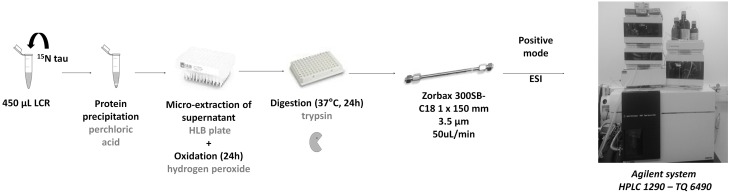
**Sample extraction: ^15^N recombinant tau was spiked on CSF and proteins were then precipitated**. Remaining proteins including tau in the supernatant were extracted on HLB cartridges. During extraction, an oxidation step was performed to oxidize methionine. Extracts were dried and then enzymatically digested by trypsin. Proteotypic peptides of tau protein were then quantified in the sample by LC-MS/MS.

Sample extraction of tau peptides were performed according to Barthelemy et al. (Barthelemy, under review). Briefly, 450 μL of CSF or 0.5% serum samples were mixed with 50 μL of ^15^N-tau-441 (50 ng/mL) in a LoBind tube. Protein precipitation was performed by adding 25 μL of 70% PCA. Samples were then vortexed, kept on ice for 15 min before centrifugation at 17,000 g at 4°C during 15 min to obtain a clear supernatant. Supernatants were acidified with 50 μL of 1% trifluoroacetic acid (TFA). SPE with a hydrophilic-lipophilic balance SPE 96-well plate was conditioned with 300 μL of MeOH and equilibrated with 500 μL of 0.1% TFA. Samples were loaded and washed with 500 μL of 0.1% TFA. For protein oxidation, 500 μL of 3% FA and 3% H_2_O_2_ solution in water was loaded on cartridge and kept 12 h at 4°C. Thereafter, cartridge was washed with 500 μL of 0.1%TFA. Oxidized tau proteins were eluted with 100 μL of 35% acetonitrile 0.1% TFA. Extracts were evaporated to dryness with a Speedvac instrument from LabConco (Kansas City, MO, USA) and resuspended with 40 μL of 1 ng/μL trypsin solution in 50 mM ammonium bicarbonate. The digestion was performed for 24 h at 37°C on a Thermomixer R from Eppendorf (Hambourg, Germany) and stopped with 5 μL of 10% FA and stored at −20°C prior to LC-MS/MS analysis (Figure 1).

#### LC-MS/MS

##### Liquid chromatography (LC) separation

LC separation was carried out on a 1290 LC system (Agilent technologies). Separation was performed with a reversed-phase Zorbax 300 SB-C18 column maintained at 60°C. The mobile phases consisted in (A) 0.1% FA in water and (B) 0.1% FA in MeOH. After an isocratic step of 2 min at 2% B, a linear gradient from 10 to 70% B was run over the next 13 min with a flow rate of 50 μL/min. The column was then washed for 1 min with 90% B and re-equilibrated during 5 min with 2% B. Eluent flow before 2 min and after 15 min was discarded with a divert valve to reduce contamination of the mass spectrometer.

##### MS/MS analysis

Mass spectrometric detection was performed using a 6490 triple quadrupole with an ESI source operating in positive mode and in dynamic MRM mode (Agilent technologies, Waldbronn, Germany). The control of the LC-MS/MS was done with MassHunter Software (Agilent technologies, Waldbronn, Germany). The ESI spray was set up according to the following settings: capillary tension 2500 V, gas flow 16 L/min with temperature of 140°C, sheath gas flow 7 L/min with temperature of 250°C, nebulizer 40 psi. Precursor ions were transferred inside the first quadrupole with high pressure ion funnel RF set to 150 V and low pressure in funnel RF set to 110 V. Collision energies (CE) and cell accelerator voltages (CA) were optimized for the peptide transitions of interest. The Skyline 2.6 version was used to conduct data treatment. All transitions per peptide were used as quantifiers and were automatically detected on specific retention time windows. LC-MS/MS were repeated 5 times for analytical validation.

#### Method validation

The calibration curve was established by linear regression and its linearity was validated according to the criterion of a Pearson correlation *r*^2^>0.99. The Limit of Quantification (LOQ) was defined as the concentration of the lowest calibration point with the relative standard deviation (RSD) of the area ratios ^14^N tau peptide/^15^N tau peptide was less than 20%. Absence of memory effects was tested by re-analyzing the calibrator point without adding any ^14^N recombinant tau protein in the end of the analytical sequence.

The intermediate precision of the entire protocol was evaluated by preparing and measuring the 20 ng/mL calibration standard over 6 different days. LC-MS repeatability was tested on the 20 ng/mL calibration standard by injecting it 4 times in a row. RSD was calculated from the area ratios ^14^N tau peptide/^15^N tau peptide.

Recovery was evaluated by analyzing human CSF sample with LTC spiked to a final concentration of 6.6 ng/mL of ^14^N tau. Recovery was calculated by applying the following equation: Recovery (%) = (Measured added concentration/Theoretical added concentration) × 100.

## Results

### Method development, linearity, and recovery

**Figure 2:**
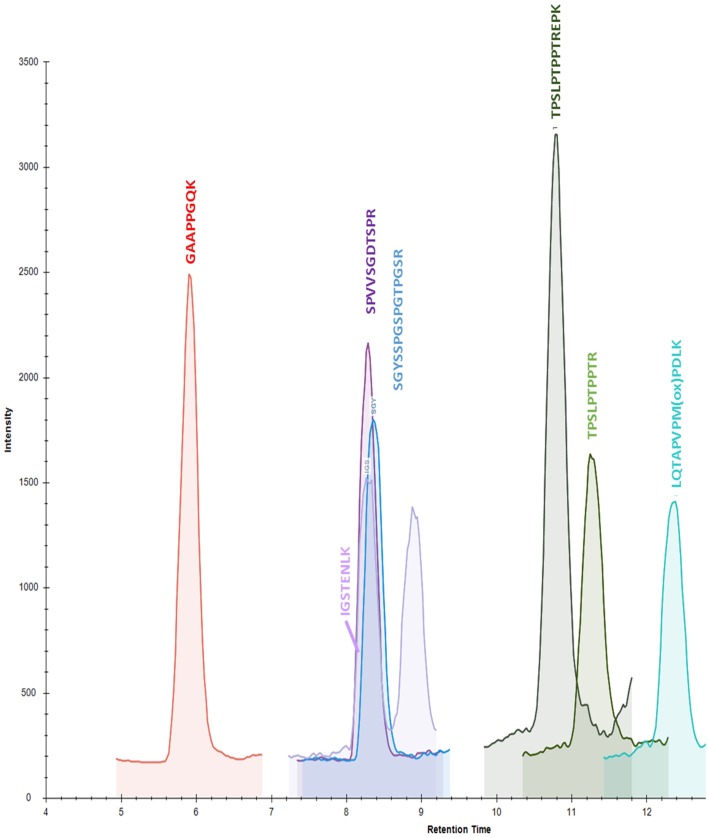
**Chromatogram of calibration standard at 32.1 ng/mL in 0.5% normal goat serum after sample preparation**.

Starting from 22 validated peptides of CSF tau protein obtained with high resolution MS in PRM mode (Barthelemy, under review), 7 peptides (GAAPPGQK, SGYSSPGSPGTPGSR, TPSLPTPPTREPK, TPSLPTPPTR, LQTPVPMPDLK, IGSTENLK, SPVVSGDTSPR) were validated using triple quadrupole in MRM mode (Table 1). One precursor ion and 2 products ion transitions were selected for the 22 peptides previously validated in PRM. We kept the most intense precursor ions (doubly charged) after optimization of the CE. Each transition was verified using Skyline Software. For the selection, three parameters were considered: signal intensities, presence of interferences and concentration in CSF pools (Table 1). The seven validated peptides (Figure 2) in human CSF had repeatable retention times with a mean RSD of 1.53% (Table 2).

**Table 1:** **Peptide selection: starting from 22 validated peptides of CSF tau protein obtained with high resolution mass spectrometry in PRM mode (Barthelemy, under review), the 7 quantifed peptides (GAAPPGQK, SGYSSPGSPGTPGSR, TPSLPTPPTREPK, TPSLPTPPTR, LQTPVPMPDLK, IGSTENLK, SPVVSGDTSPR) were selected by taking into account the three following parameters: signal intensities, presence of interferences, and concentration in CSF pools**.

**Peptide sequence**	**Peak intensity**	**Interfered**	**Increasing concentration in CSF pools**	**MRM validation**
***GAAPPGQK***	**600**	**No**	**Yes**	**Yes**
TPPAPK	2200	No	No	No
TPPSSGEPPK	200	Yes	No	No
***IGSTENLK***	**100**	**No**	**Yes**	**Yes**
***SPVVSGDTSPR***	**100**	**No**	**Yes**	**Yes**
***SGYSSPGSPGTPGSR***	**300**	**No**	**Yes**	**Yes**
VQIINK	400	Yes	No	No
SRTPSLPTPPTREPK	0	Yes	No	No
IGSLDNITHVPGGGNK	0	Yes	No	No
ESPLQTPTEDGSEEPGSETSDAK	0	Yes	No	No
***TPSLPTPPTREPK***	**1200**	**No**	**Yes**	**Yes**
STPTAEDVTAPLVDEGAPGK	0	Yes	No	No
***TPSLPTPPTR***	**400**	**No**	**Yes**	**Yes**
QAAAQPHTEIPEGTTAEEAGIGDTPSLEDEAAGHVTQAR	0	Yes	No	No
HVPGGGSVQIVYKPVDLSK	0	Yes	No	No
TDHGAEIVYK	0	Yes	No	No
DQGGYTMHQDQEGDTDAGLK	0	Yes	No	No
LDLSNVQSK	0	Yes	No	No
QEFEVMEDHAGTYGLGDR	0	Yes	No	No
***LQTAPVP****M****PDLK***	**100**	**No**	**Yes**	**Yes**
HLSNVSSTGSIDM(diox)VDSPQLATLADEVSASLAK	0	Yes	No	No
KLDLSNVQSK	0	Yes	No	No

*Bold and italics correspond to the 7 chosen tau peptides*.

*Underline characters (M) correspond to oxidized methionine*.

**Table 2:** **Method validation summary**.

**Peptide**	**Position in the tau protein sequence**	**Linear range (ng/mL)**	**LOQ (ng/mL)**	**Mean retention time (min)**	**Repeatability RSD (%)**	**Recoveryat 6.6 ng/mL (%)**	**Measured tau concentration LTC (ng/mL)**	**Measured tau concentration MTC (ng/mL)**	**Measured tau concentration HTC (ng/mL)**
**GAAPPGQK**	156 – 163	0.3 – 32.7	0.3	5.91	1.5	117	6.6	12.5	30.3
**SGYSSPGSPGTPGSR**	195 – 209	2.0 – 32.7	2.0	8.37	0.6	154	3.6	7.6	20.9
**TPSLPTPPTREPK**	212 – 224	2.0 – 32.7	2.0	10.80	1.2	109	3.2	8.1	15.7
TPSLPTPPTR^*^	212 – 223	1.0 – 32.7	1.0	11.31	1.6	107	4.6	7.3	18.9
**LQTPVPMPDLK**	243 – 254	0.5 – 32.7	0.5	12.32	3.1	98	1	2.5	17.3
**IGSTENLK**	260 – 267	1.0 – 32.7	1.0	8.28	3.2	137	2.1	4.9	9.5
**SPVVSGDTSPR**	396 – 406	0.5 – 32.7	0.5	8.28	1.3	130	0.3	1.6	3.5

**Figure 3:**
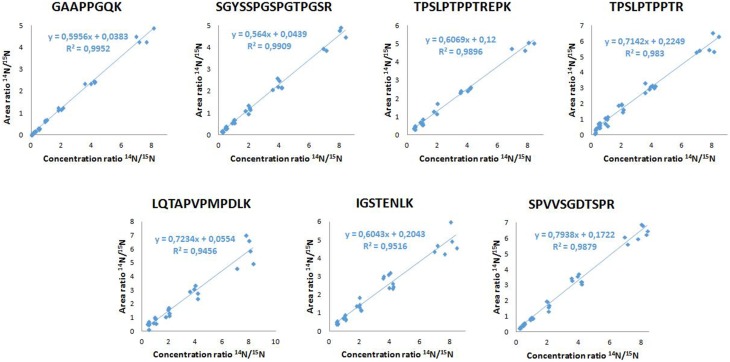
**Calibration curves of the 7 tau peptides**.

Between successive LC-MS/MS analysis, no memory effect phenomenon was observed. The calibration linearity was assessed using 10 point calibration curves of ^14^N tau standard spiked in normal goat serum 0.5%. MRM results showed linearity over 2–32.7 ng/mL concentration range (Table 2). Calibration curves were generated by linear regression analysis by plotting the peak area ratios (^14^N tau/^15^N tau) vs. concentration ratios for all measured peptides. The regression coefficients were calculated above 0.98 for the 7 considered peptides. Typical calibration curves are shown in Figure 3. The LOQs were determined over 0.3–2 ng/mL range depending on the peptide (Table 2). Calculated recovery rates were 121 ± 19% for the 7 tau peptides and using the 6.6 ng/mL calibration standard (*n* = 3). For the TPSLPTPPTR corresponding to the epitope of the ELISA capture antibody, a recovery of 107% was measured.

### Precision studies

Precision of the entire protocol (including both sample preparation and LC-MS/MS analysis) was evaluated using these samples. The RSD of the CSF pools processing was below 6% for the 7 targeted peptides. LC-MS/MS analysis was repeatable with RSD of less than 4% (*n* = 4).

### Quantification of Tau protein in CSF pools

**Figure 4:**
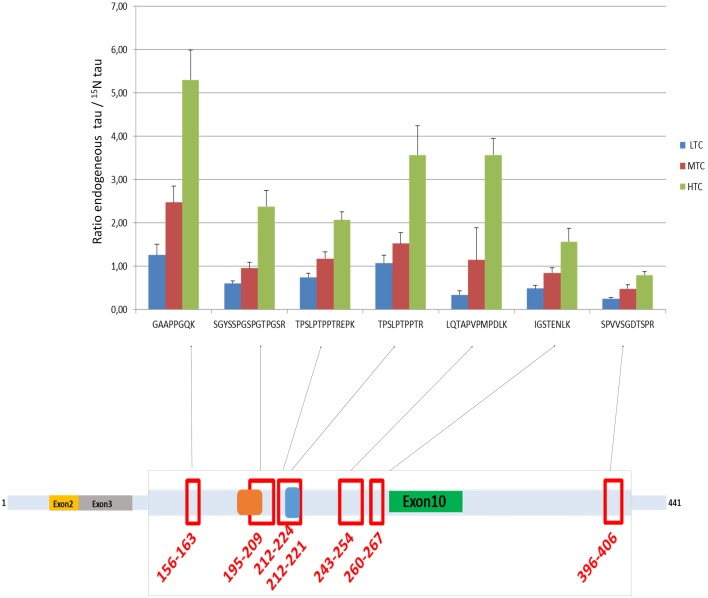
**Ratio endogenous tau/^15^N tau in the three CSF pools (LTC, MTC, and HTC) for the 7 peptides**. The scheme represents the localization of the 7 peptides on the tau protein.

Tau concentrations measured in the 3 CSF pools (LTC, MTC, and HTC) displayed different results for the 7 peptides (Table 2). For the LTC, depending on the targeted peptide, MRM-calculated concentrations ranged from 0.3 to 6.6 ng/mL, for the MTC from 1.6 to 12.5 ng/mL and for the HTC from 3.5 to 30.3 ng/mL. Calculated ratio between endogenous tau (^14^N) and tau standard (^15^N) are presented in Figure 4, showing the different concentrations obtained for each peptide in the three CSF pools. For the peptide TPSLPTPPTR corresponding to the epitope of the ELISA capture antibody, concentrations of 4.6 ng/mL for the LTC, 7.3 ng/mL for the MTC and 18.9 ng/mL for the HTC were obtained.

### ELISA quantitation and correlation with MRM

**Figure 5:**
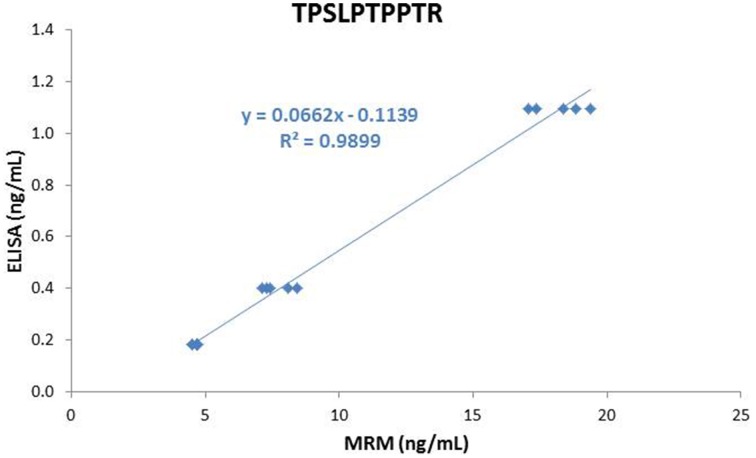
**Correlation between MRM and ELISA results**.

Tau concentration determined in the LTC, MTC, and HTC pools using ELISA were 184 pg/mL, 399 pg/mL, and 1096 pg/mL, respectively. For the 7 peptides, concentrations obtained using MRM were highly correlated with those measured by ELISA (*r*^2^ above 0.99) (see Figure 5 for the TPSLPTPPTR peptide as an example). However, ELISA concentrations were 17–25 times lower than those measured by MRM.

## Discussion

In this work, we presented for the first time an MRM based multiplex assay for tau in the CSF that did not necessitate any immuno-capture. Thanks to an adaptation of the “protein standard for absolute quantification” (PSAQ) approach (Picard et al., 2012), to an original two step purification protocol, and to the latest generation of triple quadrupole MS analyzers, we realized the tour-de-force of quantifying in parallel 7 proteotypic peptides of the tau protein. Previous MS attempts to measure tau in the CSF of patients were in fact limited to a few peptides and/or rely on immuno-precipitation procedures that are potentially subject to cross-reactivity and difficulty to obtain reproducible results when using different batches of antibodies (Portelius et al., 2008; McAvoy et al., 2014). Our method was successfully applied to the analysis of CSF pools with different levels of tau protein. Based on previous data obtained using the same sample preparation workflow but using targeted high resolution mass spectrometry (PRM), we validated 7 peptides using our triple quadrupole (MRM) compared to the 22 beforehand validated by PRM on a high resolution mass spectrometer. If MRM can be considered to be less performing in terms of resolution, it has multiple advantages compared to PRM. Mainly, the method development is much easier, data amount generated are lighter and the data processing is highly facilitated thanks to the Skyline software. Additionally, our method can be much more easily be transferred in a clinical environment where most popular mass spectrometers are triple quadrupoles.

The MRM technology also provides several analytical advantages compared with standard ELISA methods (Lehmann et al., 2013). MRM is known to be highly selective and specific (Lehmann et al., 2013) allowing to determine the absolute concentration of the targeted protein, provided that appropriate calibration standards are available. The MRM absolute quantitation of the target protein takes benefit of the advantages of isotope dilution MS (Huillet et al., 2012). Adding a known amount of an isotopically labeled internal standard at the beginning of sample preparation protocol makes it possible to account for potential material non-recovery during sample preparation, which results in better accuracy and precision. MRM assay thus showed robust pre-analytical and analytical precision, matching current clinical needs.

Interestingly, the value obtained for the quantification of tau between the two approaches (MRM vs. ELISA) showed that the concentration measured with our MRM-MS assay with the TPSLPTPPTR peptide was around 17–25 times higher than that with the ELISA test. This result is in modest agreement with the work of McAvoy et al. (2014) who had found a correlation slope of 1.8 between MSD and their IA-MS method using the same peptide. The differences between the two LC-MS/MS approaches are probably due to the different sample preparation techniques (protein precipitation followed by SPE vs. immunoaffinity) and the different standards used to establish the calibration curves. The striking difference observed between LC-MS/MS and ELISA results raises the question of what method is the most accurate. However, a first evidence in favor of LC-MS/MS is that our results were in pretty good agreement with those published in McAvoy et al.: despite different sample preparation procedures and different calibration standards were used, the 2 studies shown that for Tau concentrations below 500 pg/mL, immunoassays are negatively biased against LC/MS. Another evidence that immunoassays underestimated tau concentration is that the concentration of our protein standard was 20 ng/mL when measured by amino acid analysis, 21.4 ng/mL by LC-MS/MS and around 2 ng/mL by ELISA (data not shown). However, we didn't check that the buffer in which standards were dissolved (ammonium bicarbonate 50 mM) is compatible with Innogenetics ELISA. It can't be ruled out that standards and even our 3 CSF pools were not commutable for ELISA tests, thereby introducing matrix effects that could explain the very large discrepancy between LC-MS/MS and ELISA results. In contrary, an argument in favor of ELISA is that there were important differences in the relative levels of the 7 measured tau peptides measured by LC-MS/MS, depending on their localization on the protein sequences; as illustrated in the Figure 4. This was observed with small variations in the different pools analyzed. This result can be explained by the strong structural heterogeneity of the tau protein. Indeed, it has many proteoforms (Smith and Kelleher, 2013): six isoforms (ranging from 352 to 441 amino acids), truncated forms and forms widely modified post-translationally by glycosylation, oxidation, and phosphorylation at more than 80 sites (Iqbal et al., 2010; Hanger et al., 2014). As phosphorylation and any other post-translational modification of tau peptides induce a mass shift that results in an underestimation of total tau concentration measured by LC-MS/MS, it could be suspected that LC-MS/MS results should have been even higher. This explains why total tau concentrations measured using different peptides were not in good agreement and suggests that total tau concentration can only be measured using peptides that are neither subject to any truncation nor post-translational modification. In this sense, the peptide GAAPPGQK appears to be the best candidate because it is short enough and it can't be phosphorylated. This hypothesis is supported by the fact that among the 7 considered peptides, total tau concentration was the highest when estimated with this peptide (see Table 2). Despite total tau concentrations estimated using all the 7 peptides are in insufficient agreement to support the use of peptides that can be phosphorylated, the results obtained with the 6 other peptides also show an excellent correlation between ELISA and LC-MS/MS, which suggest that they can have a clinical relevancy as independent biomarkers. However, to do the comparison with the 7 peptides, all phosphorylated forms should have been measured. The objective of measuring 7 peptides is not really to measure total tau concentration but rather to use the 7 peptides as independent biomarkers. As suggested in Höglund et al. (Höglund et al., 2015), having insights into tau structural characterization and providing the opportunity to simultaneously quantify several peptides whose concentration is directly proportional to that of given tau proteoforms will make it possible to discover potentially more predictive biomarkers of AD. A good example is P-Tau (181), that is known to be the most relevant and predictive proteoform of tau. This work thus illustrates the need but also the future perspectives associated with the quantification of a larger number of peptides. Especially, additional investigation using in particular MRM methods designed for phosphopeptides detection (e.g., prefractionation using titanium columns) will be needed to fully interpret our results and provide the analytical methods needed to determine which proteoforms of the tau protein are the most predictive of AD. Even if tau protein is considered as a major biomarker of AD, the protein is also increased when measured by ELISA in other tauopathies like Creutzfeldt–Jakob Disease or Fronto Temporal Dementia (Green et al., 1999; Wang et al., 2010). It will be interesting to use our new method to determine whether the multiplex quantification of the 7 tau peptides described in our study could help better differentiating pathologies with increased tau in the CSF. In any case, our MRM workflow realized without immunocapture in a clinical laboratory environment represents a major improvement to the state of the art and an interesting alternative and addition to classical ELISA. Further work on large clinical cohorts will be however needed to assess the clinical interest of this new approach.

### Conflict of interest statement

The authors declare that the research was conducted in the absence of any commercial or financial relationships that could be construed as a potential conflict of interest.

## Abbreviations

AD: Alzheimer Disease
CAV: Cell Accelerator Voltage
CE: Collision Energy
CSF: CerebroSpinal Fluid
ELISA: Enzyme Linked ImmunoAssay
FA: Formic Acid
HLB: Hydrophilic-Lipophilic-Balanced
LOQ: Limit of Quantification
MAPT: Microtubule-Associated Protein Tau
PCA: Perchloric Acid
TCA: Trifluoroacetic Acid
SPE: Solid Phase Extraction
MRM-MS: Multiple Reaction Monitoring Mass Spectrometry
PTM: Post Translational Modifications.
